# Understanding psychiatric-legal disagreements in not criminally responsible on account of mental disorder cases: a gradient boosting model perspective

**DOI:** 10.3389/fpsyg.2025.1666828

**Published:** 2026-01-06

**Authors:** Aymane Haddou, Coralie Sergerie-Dufresne, Patrycja Myszak, Stéphanie Borduas Pagé, Alexandre Hudon

**Affiliations:** 1 Department of Psychiatry and Addictology, Faculty of Medicine, Université de Montréal, Montreal, QC, Canada; 2 Collège International Sainte-Anne, Lachine, QC, Canada; 3 Department of Psychiatry, Institut Universitaire en Santé Mentale de Montréal, Montreal, QC, Canada; 4 Centre de Recherche de l’Institut Universitaire en Santé Mentale de Montréal, Montreal, QC, Canada; 5 Department of Psychiatry, Institut National de Psychiatrie Légale Philippe-Pinel, Montreal, QC, Canada; 6 Groupe Interdisciplinaire de Recherche sur la Cognition et le Raisonnement Professionnel, Université de Montréal, Montreal, QC, Canada; 7 Institut de la Valorisation des Données, Montreal, QC, Canada

**Keywords:** forensic psychiatry, machine learning, not criminally responsible (NCRMD), commission d’examen des troubles mentaux (CETM), judicial decision-making, violence, artificial intelligence, mental health

## Abstract

**Background/objectives:**

According to the Canadian Criminal Code, when a court or a mental health review board makes a disposition for an individual found not criminally responsible on account of mental disorder (NCRMD), it must consider several factors: foremost, the safety of the public, as the paramount concern, as well as the mental condition of the accused, their reintegration into society, and their other needs. While psychiatric evaluations are central to these hearings, the CETM does not always follow the psychiatrist’s recommendations. This study aims to identify variables that predict agreement or disagreement between psychiatric recommendations and CETM decisions, using machine learning to better understand this decision-making process.

**Methods:**

We retrieved all CETM judgments from 2023 (*N* = 1,327) from the publicly accessible SOQUIJ database. Cases were included based on NCRMD status and judgment type (initial or annual reviews). A coding framework was developed to extract sociodemographic, clinical, legal, and administrative variables. A CatBoost classification model with SMOTE oversampling was applied to predict psychiatrist–tribunal agreement versus disagreement. Model performance was evaluated using accuracy, precision, recall, F1 score, and AUC. SHAP (SHapley Additive Explanations) values were used to assess variable importance.

**Results:**

The CatBoost model achieved an overall accuracy of 82% and an AUC-ROC of 0.672. The model performed better in identifying agreements (precision: 0.83, recall: 0.98) than disagreements (precision: 0.50, recall: 0.10). SHAP analysis revealed that the most influential predictors of agreement were whether the psychiatrist’s recommendation aligned with the CETM’s previous decision, the presence of high-risk elements, and requests for unconditional release by legal counsel.

**Conclusion:**

Our findings suggest a pattern of judicial path dependence and risk aversion in CETM decisions. Machine learning offers a promising avenue to elucidate decision-making in forensic psychiatric tribunals.

## 1 Introduction

The junction between mental health and violence has often found itself in the center of polemics. Given the several high-profile cases in recent years, public discourse quickly shifted to portraying individuals with mental illness as dangerous. However, a simple look at empirical evidence on the topic seems to reveal a more nuanced reality. For example, despite it being true that certain mental health conditions such as schizophrenia and bipolar disorder may be associated with an increased risk of violent behaviors (Whiting et al., 2022), this increase in violence is often better explained by other factors such as a diagnosis of substance use disorders (Whiting et al., 2020; Fazel et al., 2010). A systematic review of violence risk factors among individuals with psychosis also found that the two strongest predictors of future violence were prior criminal behavior and substance use, rather than the psychosis itself, which further supports this association (Lagerberg et al., 2025). These findings are also mirrored in American data from the National Epidemiologic Survey on Alcohol and Related Conditions (NESARC-III), in which the highest odds ratio for violence was once again a diagnosis of drug use disorder (Moore et al., 2021). On the other hand, it is often less known that individuals who suffer from psychiatric disorders are much more likely to be victims of violence. A Danish registry study demonstrated that these individuals had elevated risks of both being subjected to and perpetrating violence (Sariaslan et al., 2020). In certain subgroups, such as women with mental illness, the risks of victimization were even higher than those of perpetration (Dean et al., 2024). Despite literature clearly indicating that mental illness in a vacuum is a weak predictor of violence, stigmatization persists (Ahonen et al., 2017; Green, 2020).

In Québec, the commission d’examen des troubles mentaux (CETM) is a division within the Section des affaires sociales of the Tribunal administratif du Québec (TAQ). It is responsible for reviewing cases of accused found not criminally responsible on account of mental disorder (NCRMD) or unfit to stand trial. The CETM’s mandate is to find a balance between public safety, which is its main priority, and the rights and needs of the individual (Tribunal administratif du Québec, 2023).

The CETM’s possible decisions are the following: an absolute discharge, a conditional discharge, a detention with conditions, or a strict detention. As set out in Article 672.54 of the Canadian Criminal Code, these decisions aim to protect the safety of the public while imposing the least restrictive conditions necessary on the individual and ensuring the accused’s social reintegration. From 1992 to 2004, 6,802 Canadians were found NCRMD (Ministère de la Justice Canada, 2017). The National Trajectory Project (NTP) found that individuals who are NCRMD are typically single males with a psychotic disorder who received governmental income support at the time of their offense. As for the crimes committed, only a minority of the offenses were serious violent offenses. Recidivism rates are also lower in NCRMD cases compared to those found in criminally responsible individuals. However, as highlighted in Part 3 of the NTP, important interprovincial differences exist in the trajectories of NCRMD individuals. After five years, approximately 20% of people in Québec remained under the purview of a review board, compared to nearly 60% in Ontario, while the proportion of detained individuals at five years was about 5% in Québec versus almost 45% in Ontario. These variations reflect distinct legal frameworks and clinical practices across provinces. Accordingly, the present study’s findings regarding psychiatrist–tribunal agreement should be interpreted within the Québec context, where shorter detention durations and earlier reintegration are the norm (Crocker et al., 2015).

Although hearings systematically rely on detailed psychiatric reports, disagreements between the CETM and the psychiatrist’s recommendations are not uncommon (and are, to some extent, an inherent and desirable feature of the tribunal’s role) (van Es et al., 2020). The CETM is mandated to act as an independent adjudicative body that weighs clinical, legal, and public safety considerations. Complete alignment between psychiatric opinion and judicial outcome would suggest a lack of scrutiny rather than procedural harmony. Accordingly, a proportion of disagreements reflects legitimate judicial oversight, including cases where the tribunal may favor the position of the patient or the broader principle of least restrictive disposition (Kumasaka and Gupta, 1972). The present study therefore does not interpret disagreement as an error but seeks to identify the factors that statistically predict when and why such divergences occur.

By analyzing the full body of CETM decisions from the year 2023, this study investigates whether certain factors can predict agreements or disagreements between the psychiatrist’s recommendations and the CETM’s decision. This may clarify the commission’s decision-making and provide an explanation for the discrepancies found between psychiatrists and the court. Our hypothesis is that certain elements like histories of recurring violent offenses, the presence of psychotic symptoms, and less years under the CETM, will be associated with more disagreements. On the opposite end, good clinical evolution and therapeutic alliance, low perceived dangerousness and insight into the illness are expected to correlate with more agreements.

## 2 Methodology

### 2.1 Search strategies

The CETM judgements for this research project were collected using the Société québécoise d’information juridique (SOQUIJ), which is the public body responsible for compiling judicial and administrative decisions rendered in Québec. Because the CETM decisions are publicly accessible through the SOQUIJ database, no ethics review board approval was required, in accordance with Article 2.2 of the TCPS 2 (Tri-Council Policy Statement: Ethical Conduct for Research Involving Humans).

We retrieved all the decisions issued by the TAQ from 1st January 2023 to 31st December 2023. It is important to note that the SOQUIJ database is not complete and that certain judgments may be missing.

### 2.2 NCRMD decisions eligibility criteria

Given that the judicial decisions found in the TAQ section of the SOQUIJ are not exclusively NCRMD decisions, eligibility criteria were established. First, only first hearings and annual reviews held between 1st January 2023 and 31st December 2023 were retrieved. Second, judgments had to be written in French or English. Third, any decisions about fitness to stand trial or court-ordered hospital detention were excluded.

### 2.3 Data extraction

All of the CETM judgments were extracted according to the eligibility criteria mentioned above and categorized by date and outcome (unconditional discharge, conditional discharge, detention with conditions or strict detention). A coding framework was then developed to extract pertinent data from the judgments, based on sociodemographic, administrative, clinical, legal, and decisional factors.

To establish a preliminary coding grid, a sample of 20 judgments was randomly selected and independently analyzed by two investigators to ensure inter-rater reliability. The final coding grid is presented in Table 1.

**TABLE 1:** Coding grid.

Categories	Information to collect
Medico-administrative	Patient present at the hearing? (yes, no)
	Patient represented by a lawyer? (yes, no)
	If represented, is it legal aid? (yes, no)
	Presence of family/friend? (yes, no)
	Under the review board since when? (in years)
	For what reasons? (criminal code article)
	What was the verdict at the previous hearing?
	Patient’s lawyer’s request(s) for this hearing
Sociodemographic data	Sex (H, F, other)
	Age (in years)
	Children? (yes, no)
	Lives alone? (yes, no)
	Family/involvement of relatives? (yes, no)
	Homelessness? (yes, no)
	Employment? (yes, no)
	Hospitalized? (yes, no)
	If yes, for how long? (in days)
	Known to psychiatry for how long? (in years)
	History of previous hospitalizations? (how many)
	History of previous incarcerations? (how many)
	Police involvement in the last year? (yes, no)
Relevant clinical history	Primary diagnosis?
	Other diagnoses mentioned
	Lifestyle habits: tobacco? (yes, no)
	Lifestyle habits: alcohol? (yes, no)
	Lifestyle habits: cannabis? (yes, no)
	Lifestyle habits: stimulants? (yes, no)
	Lifestyle habits: other (specify)?
	Medical history
	Medication adherence/compliance? (yes, no)
	Attends follow-up appointments? (yes, no)
Symptoms and signs in the last year	Auditory hallucinations (yes, no)
	Visual hallucinations (yes, no)
	Other hallucinations (specify)
	Paranoid delusion (yes, no)
	Persecutory delusion (yes, no)
	Grandiose delusion (yes, no)
	Somatic delusion (yes, no)
	Erotomanic delusion (yes, no)
	Other delusion (specify)
	Thought disorder (specify)
	Aggressiveness (yes, no)
	Self-directed violence (last year, specify if physical or verbal)
	Other-directed violence (last year, specify if physical or verbal)
	Insight (good, partial, poor, absent)
	Judgment (good, partial, poor, absent)
Judicial history	Relevant history (criminal code article)
	In the last year (theft)? (yes, no)
	In the last year (break-in)? (yes, no)
	In the last year (drug sales/trafficking)? (yes, no)
	In the last year (runaway)? (yes, no)
	In the last year (non-compliance with rules)? (yes, no)
	In the last year (assault: if yes, specify if armed)? (yes, no)
Verdict	Request from the treating team (detention/release with/without conditions)
	Risk factors considered by the team (low, moderate, high)
	Verdict of the CETM (detention/release with/without conditions)
	Delegation of authority (yes, no)
	Agreement between psychiatrist’s request and verdict (yes, no)

**FIGURE 1:**
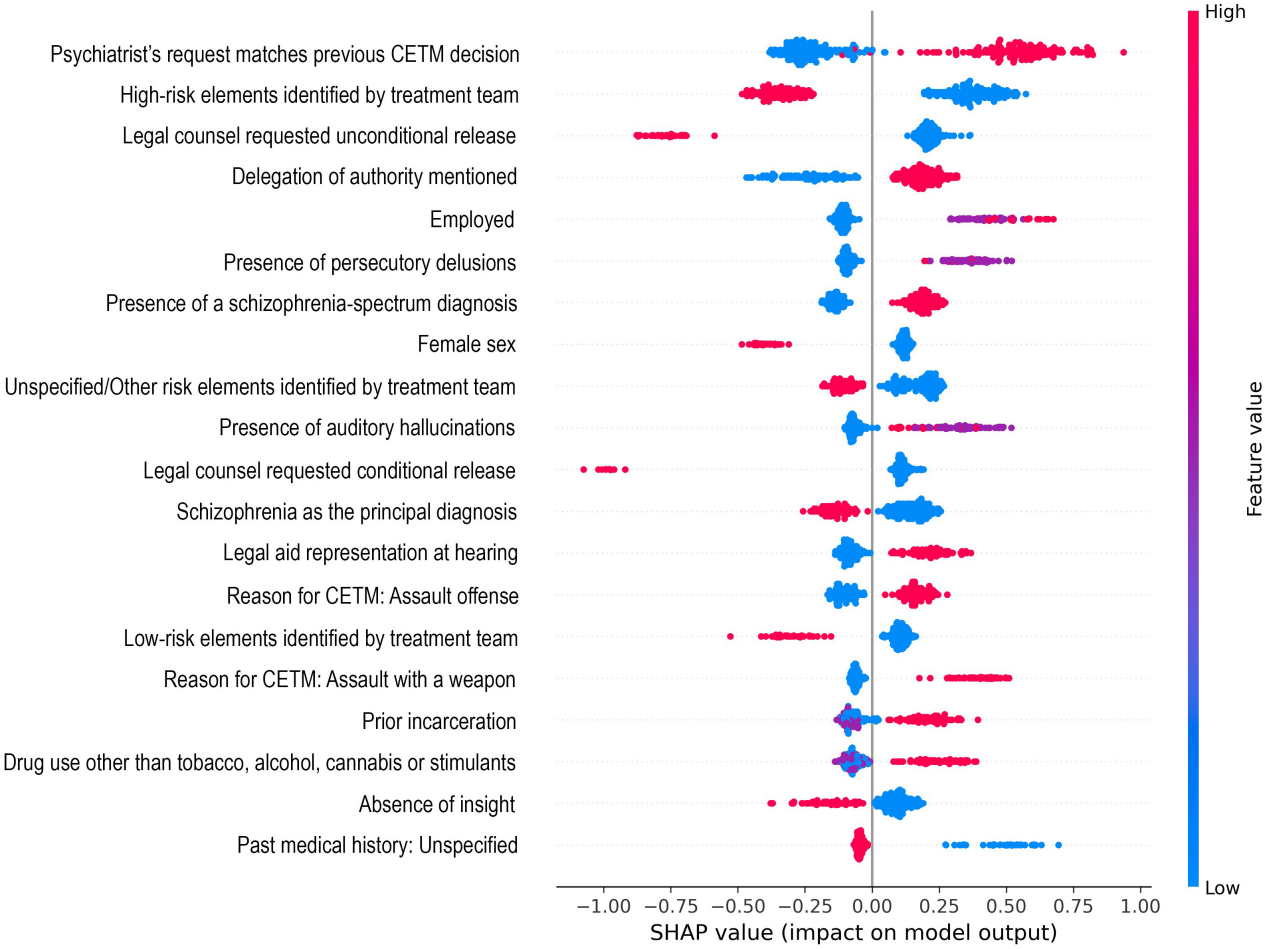
SHAP summary plot of variables’ contribution to the model’s predictions.

After obtaining this preliminary grid, a second random sample of 10 judgments was independently analyzed by the same investigators to test the reliability of the coding grid. The final coding grid used for the full analysis is presented in Figure 1.

### 2.4 Data analysis

Using the final coding grid mentioned above, all the identified variables were collected from each judgment. Everything was then compiled into a single structured document. The outcome variable was defined as either agreement or disagreement between the psychiatrist’s recommendation and the Commission’s decision. After excluding incomplete cases that could not be analyzed, a total of 1,327 judgments remained. A CatBoost classification model (version 1.2.8) was used for the analysis. CatBoost is a machine learning algorithm that uses gradient boosting. This method works by creating an initial decision tree that gets optimized through the sequential addition of small decision trees called weak learners. Each new weak learner improves on the preceding model by aiming to correct the errors. This is done by using a gradient descent to minimize the loss function. This model was selected because the dataset had a mix of categorical, numerical, and binary variables, which CatBoost is able to handle natively without the need of data preprocessing. The algorithm also performs well with imbalanced and smaller dataset, which are both present in the current data table.

The data was randomly separated using an 80/20 split for training and testing sets. Because our data was imbalanced due to it having significantly more agreements than disagreements, we used SMOTE (Synthetic Minority Oversampling Technique) to artificially increase the disagreements in the training set. SMOTE is a statistical technique used to improve a model learning’s capacity on an imbalanced dataset. It generates new synthetic examples of the minority class by imputing new data points based on the feature similarities between real minority class instances and their nearest neighbors. The following settings were used to train our model: iterations = 300, learning rate = 0.05, depth = 7, random state = 42, verbose = 0, and class weights = [1, 1].

We then evaluated our model’s performance by calculating the accuracy, precision, recall, F1 score and AUC (Area Under the Curve) on the test set. SHAP (SHapley Additive Explanations) values were then computed to assess the contribution of each variable on the final prediction. Cross-validation was also performed by using other gradient boosting models such as XGBoost and LightGBM to assess the consistency of the results.

It is important to note that the model and analyses were based solely on written judgments and psychiatric reports obtained from the SOQUIJ database. In Québec, CETM hearings also include oral testimonies, cross-examinations, and real-time discussions between parties, which are not transcribed in these public documents. These oral exchanges may influence decision-making through credibility assessments, clarifications of risk interpretation, or evidentiary nuances that are not captured in written form. Consequently, while the model identifies patterns within the documentary record, it does not account for performative, interactional, or contextual dynamics that may also contribute to tribunal outcomes.

## 3 Results

### 3.1 Model performance

A CatBoost classifier was trained on the processed dataset. SMOTE was applied to the training sample given the class imbalance between agreements and disagreements. The model’s performance was then evaluated on the test sample of 277 cases, which represented 20% of our dataset. The model achieved a classification accuracy of 82% and an AUC-ROC of 0.672. It also had a weighted precision of 0.77, a weighted recall of 0.82 and a weighted F1-score of 0.77. A breakdown of performance by class is presented below in Supplementary Figure 1. Model performance metrics are presented in Table 2 as well as the confusion matrix of the classifier predictions on the test set in Table 3.

**TABLE 2:** Model performance metrics on test set for predicting agreements or disagreements between psychiatrists’ recommendations and the CETM’s decisions.

Class	Precision	Recall	F1-score	Support
Disagreement	0.50	0.10	0.17	50
Agreement	0.83	0.98	0.90	227
Accuracy			0.82	227
Macro Avg	0.67	0.54	0.53	227
Weighted Avg	0.77	0.82	0.77	227

**TABLE 3:** Confusion matrix of CatBoost classifier predictions on test set.

Actual results	Predicted: disagreement	Predicted: agreement
Actual: disagreement	5	45
Actual: agreement	5	222

The model’s class-level performance was higher for agreements compared to disagreements. This can be seen when comparing the precision, recall and F1-score in both classes. Precision measures the proportion of true positives among all positively predicted cases. This translates to the model correctly classifying 222 judgements as agreements and 5 as disagreements, out of the 267 agreement predictions and 10 disagreement predictions.

Recall, also known as the true positive rate, represents the proportion of actual positives that were correctly classified. Our model correctly identified 5 out of 50 disagreements and 222 out of 227 agreements, resulting in a recall of 0.10 and 0.98, respectively.

As for the F1-score, it offers a more balanced performance metric that considers class imbalance. Unlike accuracy, which simply represents the proportion of correct predictions (0.82), the F1-score (0.77) is the harmonic mean of precision and recall. It balances these two metrics while placing an emphasis on lower values, in our case being the performances in the disagreement class.

### 3.2 Variable importance

SHAP values were used to interpret the model and assess the impact of the variables on the prediction. The SHAP summary plot (Figure 1) displays the distribution of variables and their impacts across all test judgements. A positive SHAP value in the summary plot indicates that the variable in question pushes the model toward an agreement, compared to a negative value which indicates a disagreement. As for the color used in the plot, red indicates the presence of the variable, blue the absence, and purple is unavailable information.

The factor with the most weight in the prediction was *Psychiatrist’s request matches previous CETM decision*, which indicates whether the psychiatrist’s current recommendation corresponds with the CETM’s previous verdict. This feature had the highest mean absolute SHAP value (SHAP = 0.38). The SHAP Summary Plot shows that when a recommendation matches the previous CETM decision, it nearly always pushes the algorithm toward an agreement, while novel recommendations tend to predict a disagreement. The second most influential variable was *High-risk elements identified by treatment team* (SHAP = 0.36). Judgments were categorized as high, medium, or low risk based on explicit references in the final report; in the absence of a clear risk assessment, they were classified as other/unspecified. When high-risk elements were present, this factor seemed to tilt the prediction toward disagreement. In contrast, the absence of any high-risk elements shifted the outcome in the direction of agreement. The third factor in importance was *Legal counsel requested unconditional release* (SHAP = 0.28), meaning that the defendant’s lawyers asked for the complete release of the individual from the CETM. A request of the sort heavily pushed the model toward disagreement. Variables with the highest impact on the model output based on SHAP values are presented in Supplementary Table 1.

## 4 Discussion

In this study, by thoroughly analyzing all the CETM’s NCRMD judgments, we aimed to identify factors that could predict agreements or disagreements between the psychiatrists’ recommendations and the final decision of the court. To extract these different variables, a CatBoost classifier, which is a type of machine learning algorithm, was applied on the 1327 CETM judgements from 2023. To assess the model’s performance, multiple performance metrics were obtained. The AUC-ROC of 0.672 indicates a moderate ability of the algorithm to distinguish agreements and disagreements. The precision, recall and F1-score were all significantly higher in the agreement class compared to the disagreements. This reflects that the model identified well agreements but struggled with disagreements. This notable difference is likely due to the class imbalance in our data. Given that most of the CETM’s judgments are classified as agreements, our model was mostly trained on this class. Using SMOTE, which synthetically increased the disagreement group, resulted in limited benefits. By computing SHAP values, we were able to assess the contribution of each variable on the model’s prediction. The factor with the highest importance was whether the psychiatrist’s recommendation matched the CETM’s previous decision (SHAP value = 0.38). Other highly influential variables on the model’s predictions were the presence of high-risk elements, and whether legal counsel requested an unconditional release.

As mentioned above, the most important factor was whether the psychiatrist’s recommendation matched the CETM’s previous decision. As seen in the SHAP Summary Plot, the presence of an alignment in decision-making pushes the model toward an agreement. However, a new recommendation from the psychiatrist that diverged from the CETM’s latest decision clearly influenced the algorithm in predicting a disagreement. This finding suggests a pattern of path dependence in the CETM’s judgments, where past decisions hold a greater influence and limit future decision-making. This tendency is corroborated in studies of mental health tribunals. In Peay’s (1981) *Mental Health Review Tribunals*, it was found that courts tend to default to a *status quo* decision, especially in cases of detention, only diverging from it in the presence of major evidence of changes. With the 2018 UK *Final report of the* <ref> Independent Review of the Mental Health Act 1983 (2018), we gain a better understanding of this path dependence, which appears to still be present 40 years after Peay’s *Mental Health Review Tribunals*. According to the report, only 8.6% of tribunal hearings resulted in discharge, with the driving factors being fear of accountability and risk-aversion. Making a novel decision comes with an increased perception of risk, where the repercussion of a potential faulty judgement could be irreparable. Such pressure encourages reliance on a precedent judgement as a safety net. This continuity, which was shown in the literature and in our model’s prediction, begs the question about a potential bias of the court toward consistency rather than adapting decisions to the clinical evolution of patients. However, the *Mental Health Review Tribunals* in England and Wales function within a different legal framework, patient population, and procedural mandate than Canada’s CETM. In the UK, patients under forensic orders cannot be discharged without conditions, and the tribunal composition, party roles, and discharge mechanisms differ substantially from the Canadian NCRMD context. Moreover, the legislative landscape in England and Wales was reformed following Peay’s, 1981 study, including through the 1983 and subsequent amendments to the Mental Health Act. As such, the comparison is meant to highlight a general tendency toward path dependence across jurisdictions, rather than imply direct equivalence between legal systems.

The second most impactful variable was the presence of high-risk elements identified by the treatment team. Interestingly, when high-risk elements were mentioned in the forensic evaluation, it often led toward disagreements between the court and the psychiatrist. This aligns with the CETM’s stated mandate of prioritizing public safety. Prior research has shown that courts tend to adopt a more risk-averse position when dangerousness risk factors are mentioned, despite the presence of therapeutic stability and/or improvement (McSherry et al., 2006). This is further explained by Wexler in *Mental Health Law: Major Issues* (Wexler, 1981), where it was revealed that institutional decision-making often gravitated around the topic of high-risk features, often at the expense of therapeutic and clinical considerations. He also noted that courts tended to expand the definition of dangerousness to justify stricter judgments. On the other hand, when there was no mention of high dangerousness, the algorithm interpreted it as a factor associated with agreements. This would indicate that, in the absence of red flags, the psychiatrist’s clinical evaluation seems to carry more weight in the eyes of the tribunal.

The third most important factor was the request for an unconditional release by the legal counsel. When the defense’s lawyer made such a request, the algorithm seemed to lean toward disagreement. However, the absence of an unconditional release demand was strongly associated with agreement. Interestingly, this pattern was also present, to a lesser extent, with the variable *Request for a Conditional* release. It is also important to note that in 75.9% of cases, legal counsel and psychiatrist had similar recommendations, with 64.5% being a joint request for release. Given the courts risk-aversion and tendency to relying on path dependence, it comes as no surprise that despite having both psychiatrist and legal counsel agreeing on a recommendation, release requests nearly systematically caused disagreements. This risk-aversion is further supported by the fact that any request of the counsel other than release, which is either a detention request or an absence of request, pushed the algorithm toward agreement. One possible explanation for the association between absent requests and agreements lies in the presence, or lack thereof, of legal counsel. Absence of requests often happen in cases where no legal representation is present, and research shows that it tends to lead to stricter outcomes. When comparing outcomes in defendants with and without legal representation, individuals without legal representation were less likely to attend their court hearings, resolve their charges and complete mental health court programs (Linhorst et al., 2022). In such cases, the absence of legal counsel would then leave the tribunal’s decision-making unopposed, defaulting either to the psychiatrist’s recommendation or to its own risk-averse tendency.

This study offers one of the first applications of explainable machine learning to decision-making within a forensic psychiatric tribunal. By analyzing all publicly available CETM judgments rendered in a full calendar year, it provides an unprecedented empirical overview of the determinants of agreement between psychiatric recommendations and judicial outcomes in Québec’s NCRMD system. The use of CatBoost, optimized for mixed and imbalanced datasets, allowed for robust classification across diverse variable types. Moreover, the incorporation of SHAP values enabled transparent interpretation of the model’s reasoning, bridging computational outputs with legal-psychiatric understanding. Beyond its technical contribution, the study illustrates how interpretable artificial intelligence can illuminate institutional patterns (such as path dependence and risk aversion) that are difficult to quantify through traditional qualitative or statistical methods.

### 4.1 Limitations

First, the CETM judgments obtained came from the SOQUIJ database, which is not exhaustive. Therefore, the dataset may not be necessarily reflective of all decisions rendered by the CETM. Second, due to the heterogenous nature of forensic reports, certain variables identified in the coding grid were not consistently mentioned across all judgements, which could impact the model’s performance. Third, while SHAP is an excellent tool to reveal and easily interpret associations, it is not indicative of causation. SHAP only reflects the decision-making of the model, but it cannot be directly translated to real life. Finally, given that disagreements were underrepresented in our dataset, the model’s learning capacity from this class was limited. This was reflected in the lower performance metrics compared to the agreement class. Future work could address this limitation by collecting judgments from other years to increase the sample size of disagreements.

### 4.2 Future directions

Future studies should link CETM decisions with justice and health data to follow individuals over time. In particular, it would be important to compare people who received a reduction in restrictions (for example, from detention to conditional or absolute discharge) after a disagreement between the psychiatrist and the CETM, with those where there was agreement. Such research could clarify whether disagreements are associated with higher or lower rates of recidivism, re-hospitalization, or other clinical outcomes, helping to assess the long-term impact of tribunal decision-making.

## 5 Conclusion

This study highlights how judicial decision-making within Québec’s CETM is shaped not only by clinical recommendations but also by institutional dynamics such as path dependence and risk aversion. By applying a CatBoost machine learning model to a full year of CETM judgments, we identified key variables (such as alignment with prior decisions, presence of high-risk elements, and legal counsel’s requests) that significantly influence whether a psychiatrist’s recommendation is upheld. While the model demonstrated strong performance in predicting agreements, its limited sensitivity for disagreements underscores the complexity and imbalance inherent in these legal processes. Our findings raise important questions about the weight given to precedent over clinical evolution and suggest that decisions may at times prioritize procedural continuity over individualized care trajectories. Future research should aim to improve model sensitivity for disagreement cases and expand the dataset across multiple years to validate the generalizability of these trends. This work contributes to a deeper understanding of how legal and psychiatric frameworks intersect, and occasionally diverge, in the management of individuals found NCRMD.

## Data Availability

The original contributions presented in this study are included in this article/Supplementary material, further inquiries can be directed to the corresponding author.
