# Supplementary material for: Understanding psychiatric-legal disagreements in not criminally responsible on account of mental disorder cases: a gradient boosting model perspective
Source: Front Psychol. 2026 Jan 6;16:1666828. doi: 10.3389/fpsyg.2025.1666828 (PMC12815741; doi:10.3389/fpsyg.2025.1666828)
Supplement: Supplementary file 1 [file Supplementary_file_1.docx]

Supplementary Material

# Supplementary Data

# Supplementary Figures and Tables

Supplementary Table 1 : *Variables with the Highest Impact on Model Output Based on SHAP values*

| **Variables** | **Mean SHAP Value** |
| --- | --- |
| Psychiatrist’s request matches previous CETM decision | 0.383 |
| High-risk elements identified by treatment team | 0.363 |
| Legal counsel requested unconditional release | 0.282 |
| Delegation of authority mentioned | 0.203 |
| Employed | 0.197 |
| Presence of persecutory delusions | 0.173 |
| Presence of a schizophrenia-spectrum diagnosis | 0.169 |
| Female sex | 0.156 |
| Unspecified/Other risk elements identified by treatment team | 0.151 |
| Presence of auditory hallucinations | 0.147 |
| Legal counsel requested conditional release | 0.144 |

## Supplementary Figures

Supplementary Figure 1: *SHAP Bar Plot of Variables’ Contribution to the Model’s Predictions*

**
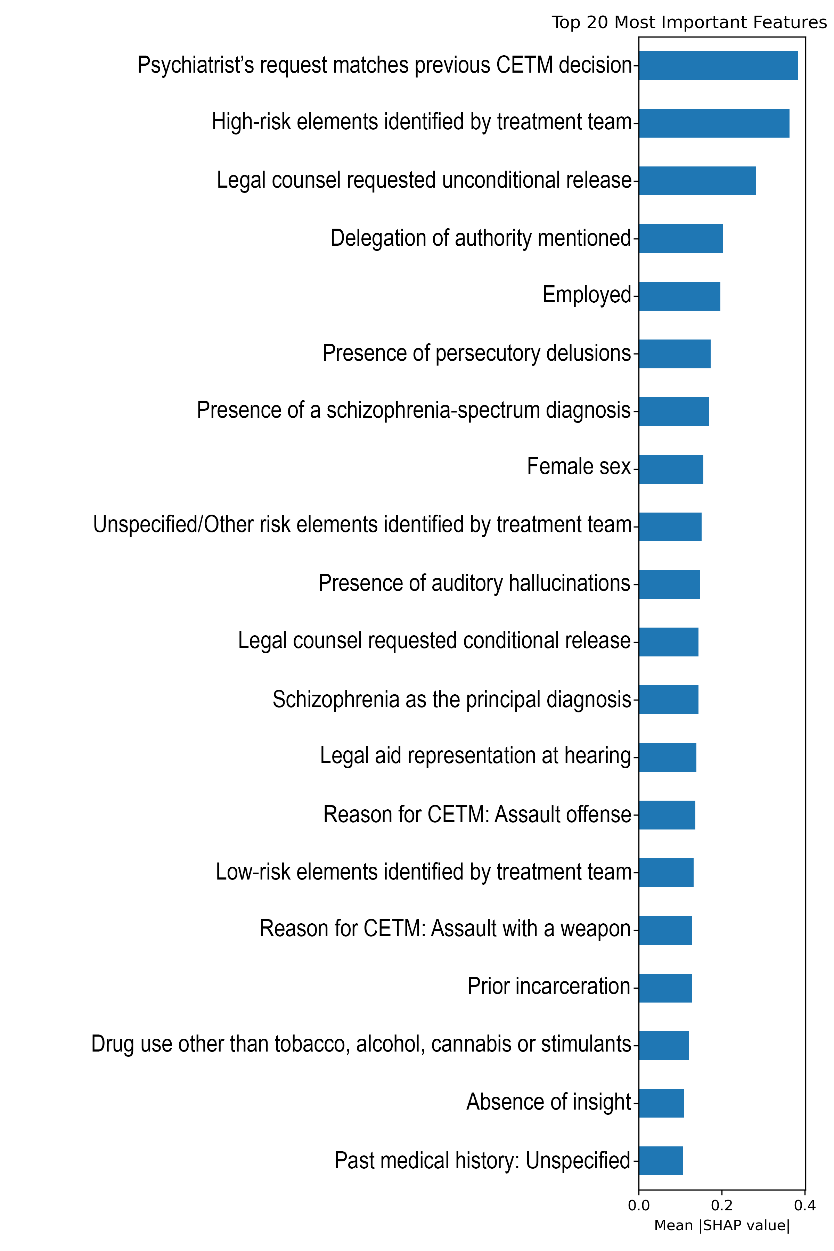
**
